# Nano-Biochar Prepared from High-Pressure Homogenization Improves Thermal Conductivity of Ethylene Glycol-Based Coolant

**DOI:** 10.3390/nano14151308

**Published:** 2024-08-03

**Authors:** Youheng Wang, Xianjun Hou, Hong Yu, Weiwei Guan, Yuxin Ma, Mohamed Kamal Ahmed Ali

**Affiliations:** 1 Hubei Key Laboratory of Advanced Technology for Automotive Components, School of Automotive Engineering, Wuhan University of Technology, Wuhan 430070, China; yhengwang@163.com (Y.W.); 15752483446@163.com (H.Y.); gww2285731495@whut.edu.cn (W.G.); mayuxin1291@126.com (Y.M.); 2 Hubei Collaborative Innovation Center for Automotive Components Technology, Wuhan 430070, China; 3 Chongqing Research Institute, Wuhan University of Technology, Chongqing 410121, China; 4 State Key Laboratory of Solid Lubrication, Lanzhou Institute of Chemical Physics, Chinese Academy of Sciences, Lanzhou 730000, China; eng.m.kamal@mu.edu.eg; 5 Automotive and Tractors Engineering Department, Faculty of Engineering, Minia University, El-Minia 61519, Egypt

**Keywords:** thermal management system, sesame stalks, dynamic viscosity, dispersion stability

## Abstract

As an environmentally friendly material, biochar is increasingly being utilized in the field of heat transfer and thermal conduction. In this study, nano-biochar was prepared from high-pressure homogenization (HPH) using sesame stalks as the raw material. It was incorporated into ethylene glycol (EG) and its dispersion stability, viscosity, and thermal conductivity were investigated. The nano-biochar was stably dispersed in EG for 28 days. When the concentration of the nano-biochar added to EG was less than 1%, the impact on viscosity was negligible. The addition of 5 wt.% nano-biochar to EG improved the thermal conductivity by 6.72%, which could be attributed to the graphitized structure and Brownian motion of the nano-biochar. Overall, nano-biochar has the potential to be applied in automotive thermal management.

## 1. Introduction

Automotive power systems are evolving towards more environmental friendliness, intelligence, and efficiency, leading to increasingly stringent requirements for thermal management systems. Nanofluids, with excellent heat transfer-enhancing capability, hold potential as a novel cooling media for automotive thermal management systems [1,2]. As reported recently, nanofluids are used as enhanced cooling media in various automotive powertrain systems, including internal combustion engines [3], lithium batteries [4], and fuel cells [5,6]. In addition, the application of nanofluid as a new cooling medium can effectively enhance heat dissipation capacity, miniaturize radiators, and reduce the energy consumption of pumps, achieving the goals of energy saving, emission reduction, and light weighting [7]. From the perspective of materials, the nanoparticles added to cooling liquids generally include nanometals [8], metal oxide nanoparticles [9,10], and carbon nanomaterials [11,12].

However, metals and metal oxides suffered from sedimentation due to their excessive density, which will largely reduce the performance of nanofluid. Furthermore, nano additives such as metal oxide nanoparticles developed for industrial applications will cause air pollution, inflammation, and skin diseases [13]. Although carbon nanomaterials like nano-graphene [14], carbon nanotubes, and graphene oxide possess low density and high thermal conductivity, the high costs limit their industrial application. Moreover, the preparation processes of graphene oxide involve the utilization of hazardous chemicals (H_2_SO_4_ and KMnO_4_) [15]. Therefore, it is imperative to develop “green” nanomaterials for coolants by considering a series of issues such as the dispersion stability of nanomaterials, costs, and environmental friendliness.

Biochar, as a burgeoning eco-friendly material, is obtained from pyrolyzing biomass in an anoxic and high-temperature environment. Typically, waste biomass is disposed of through incineration or landfill, so the development and application of biochar are significant for biomass resource utilization and carbon sequestration [16]. Biochar is mainly applied in water purification [17,18], soil amendment [19], and battery electrode materials [20]. In recent years, biochar has gained popularity as an additive to enhance the thermal conductivity of phase change materials and building materials owing to its low cost, high thermal stability, rich surface functional groups, and environmental friendliness [21,22]. Lv et al. [23] found that the biochar derived from rice husk performed well in thermal conductivity when added to different organic phase change materials. The thermal conductivity increased by 46%, 13.8%, and 27.1% compared with pure paraffin, stearic acid, and polyethylene glycol, respectively. In the application of biochar in liquid cooling media, when 5% garlic stem biochar was added to liquid paraffin wax, the thermal conductivity was improved by 7.2% compared to pure paraffin wax [24]. However, few studies have investigated the heat transfer and thermal conductivity of biochar in EG or water. This is primarily because the large size of biochar and its poor dispersion stability restricts its application in cooling fluids.

Nano-biochar has garnered growing interest owing to its smaller size, greater mobility, and ease of modification [25]. The previous study confirms the excellent dispersion stability of nano-biochar exhibited in poly-alpha olefin-6 (PAO6) liquids [26]. HPH, as a continuous, low-cost, scalable, and eco-friendly method for the preparation of nanofluids, has attracted great interest [27,28]. In the study by Melchior Sofia [29], the nano-sized liposomes (about 30 nm) were obtained by the HPH method at 150 MPa in a single-pass condition. In addition, Ma et al. successfully prepared nano-sized fibers in 20–1000 nm using an HPH method under different operation conditions [30]. 

To the best of the author’s knowledge, no work has been reported about the use of nano-biochar prepared by the HPH method as an additive in an EG cooling medium. In our study, modified nano-biochar was prepared by the HPH method and comprehensive characterization was performed to evaluate its potential as an additive in EG. The dispersion stability of the nano-biochar in EG was investigated using natural sedimentation and dynamic lighting scattering (DLS). Additionally, the viscosity/temperature characteristics and thermal conductivity of the nano-biochar coolant were studied to explore its application in cooling systems. This study is significant for waste biomass recycling, energy saving, and the application of green materials in thermal management systems.

## 2. Materials and Methods

### 2.1. Materials

Hubei Province is a significant sesame cultivation region in China. Sesame stalks are readily available in this area and are usually incinerated as agricultural waste in rural areas, contributing to atmospheric pollution. Compared to rice straw and wheat straw, sesame stalks have a lower ash content [31]. In addition, sesame stems are softer and more easily ground into powder compared to wood. Consequently, sesame stalks were selected as the raw material for the preparation of biochar. The raw materials were cleaned to remove impurities and then ground in a pulverizer. Subsequently, the powder was passed through a 200-mesh sieve and dried in a drying oven at 80 °C for 12 h. 

**Figure 1:**
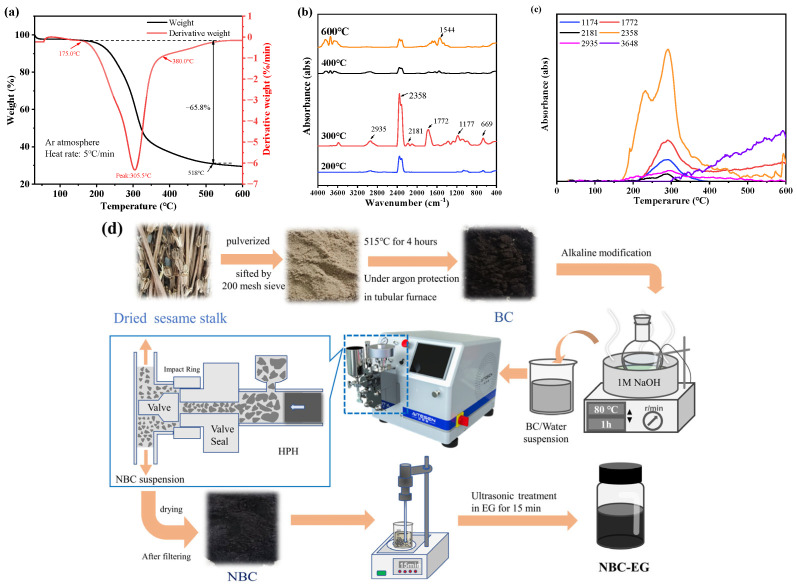
(**a**) TGA analysis of the biomass, (**b**) the FTIR spectra of the gasses obtained during pyrolysis at various temperatures, (**c**) the characteristics of typical functional groups at different temperatures, and (**d**) the preparation of NBC-EG coolant.

The pyrolysis characteristics of the sesame straw powder were evaluated using thermogravimetric-Fourier transform infrared spectrometer analysis (TGA-FTIR), the TGA result is shown in Figure 1a. The TGA experiment was conducted under an argon gas environment, with a heating rate of 5 °C/min. At the low-temperature stage (≤100 °C), the primary mass loss is attributed to the moisture in the biomass, accounting for about 2.32%. The mass loss between 100 and 175 °C is about 1%. Within 175–380 °C, the biomass powder was rapidly pyrolyzed, resulting in a 65.8% mass loss and the pyrolysis rate peaked at 305.5 °C. Subsequently, the pyrolysis gradually slowed down and essentially concluded at 518 °C. Based on the TGA curve of the sesame straw, 515 °C was selected as the temperature for the pyrolysis. The FTIR spectra of the gasses obtained during pyrolysis at various temperatures are presented in Figure 1b. The wavenumber of 3500–3900 cm^−1^ and 1544 cm^−1^ correspond to -OH, corresponding to the crystal water in biomass, while 2935 cm^−1^ is attributed to CH_4_ and hydrocarbon. The peaks at 2400–2250 cm^−1^ and 699 cm^−1^ are related to CO_2_, the peaks 2250–2000 cm^−1^ correspond to CO, and 1772 cm^−1^ corresponds to the vibration of C=O, associated with acid, aldehyde, and ketone groups. The peak at 1177 cm^−1^ is attributed to the vibration of the C-O, associated with phenol, alcohol, and ester groups [32]. At 300 °C, the higher peak intensity in the FTIR spectra confirmed a greater release of gasses and a faster pyrolysis rate. The characteristics of typical functional groups at different temperatures is shown in Figure 1c; the wavenumber of 2358 cm^−1^ shows bimodal distribution, and the peak at 232 °C corresponds to the CO_2_ released from the decomposition of hemicellulose, along with CO and CH_4_ emissions. Another peak at 293 °C is associated with the CO_2_ emission resulting from the decomposition of cellulose. The peaks of C-O, C=O, CO, and CH_4_ are all located around 300 °C, a substantial number of gasses was released at this temperature, which is in line with the TGA result. After 300 °C, the crystal water begins to evaporate. Gasses released after 380 °C are attributed to the decomposition of lignin.

The pre-dried biomass powder sample was in a capped boat-shaped crucible and put in a muffle furnace. With a continuous argon gas flow rate of 0.12 L/min, the sample was heated to 515 °C at a rate of 5 °C/min, maintained at 515 °C for 4 h, and then cooled naturally to room temperature. The product was the pristine biochar, denoted as BC. The deionized water used in the experiment was produced by a pure water machine (LD-UPW-30, Shanghai LiDing Water Treatment Equipment Co., Ltd., Shanghai, China), with a resistance of 18.25 MΩ. NaOH (96%) and EG (98%) were purchased from Aladdin Company, Bay City, MI, USA; they were all analytical grade. The purity of the argon gas used in the experiment exceeded 99.999%.

### 2.2. Preparation of Nano-Biochar

The preparation process of nano-biochar is depicted in Figure 1d. First, the pristine BC was placed in a 1 M NaOH solution (200 mL) and continuously stirred for 1 h at 80 °C for alkaline modification. Next, the sample was washed several times with deionized water using vacuum filtration until the pH became neutral. Then, the modified biochar sample was dried at 80 °C for 12 h to obtain the alkalized biochar powder.

Before the HPH process, 0.5 g of the alkalized biochar sample was mixed with 100 mL of deionized water and dispersed ultrasonically for 10 min to ensure thorough mixing of the solution. A high-pressure homogenizer (HPH-L2, Suzhou Aitesen Pharmaceutical Equipment Co., Ltd., Suzhou, China) operated at the homogenization flow rate of 250 mL/min, and the homogenization pressures were set at 40, 80, and 120 MPa separately. The homogenization time was set at 15, 30, and 60 min. After the HPH process, the solution sample was filtered and dried at 80 °C for 4 h to obtain the alkalized nano-biochar, denoted as NBC. Finally, the NBC and the EG solution were mixed under ultrasonication for 15 min to obtain the NBC-EG coolant.

### 2.3. Characterization Technology

The surface morphology of the biochar samples was analyzed under a scanning electron microscope (SEM, JFM-7500F, JEOL, Tokyo, Japan). The atomic force microscope (AFM, Dimension FastScan/Icon, Bruker, Ettlingen, Germany) was used to measure the microstructure of the biochar. The surface functional group characteristics were observed with a FTIR (ALPHA II, Bruker, Germany), focusing on the vibration of the absorption peaks in the wave number of 400–4000 cm^−1^. The chemical composition of the sample surface was analyzed under an X-ray photoelectron spectroscope (XPS, ESCALAB 250Xi, Thermo Scientific, Waltham, MA, USA), and the C1s peak was corrected to 284.8 eV to calibrate the binding energy. Brunauer/Emmett/Teller Analyzer (BET, NOVAtouch, Quantachrome, Boynton Beach, FL, USA) was used to test the surface area of the biochar. Dynamic light scattering (DLS, 90Plus PALS, Brookhaven Instrument Inc., Holtsville, NY, USA) was used to characterize the particle size distribution of the NBC sample. Each sample was measured three times and the results were averaged. The viscosity of the coolant was measured using a viscometer (DV3TLVTJ0, Brookfied, New York, NY, USA), and the thermal conductivity characteristics were detected with a thermal conductivity meter (TC3000E, Xiatech Electronics Co., Ltd., Xi’an, China) at different temperatures.

## 3. Results and Discussion

### 3.1. Characterization of Biochar

**Figure 2:**
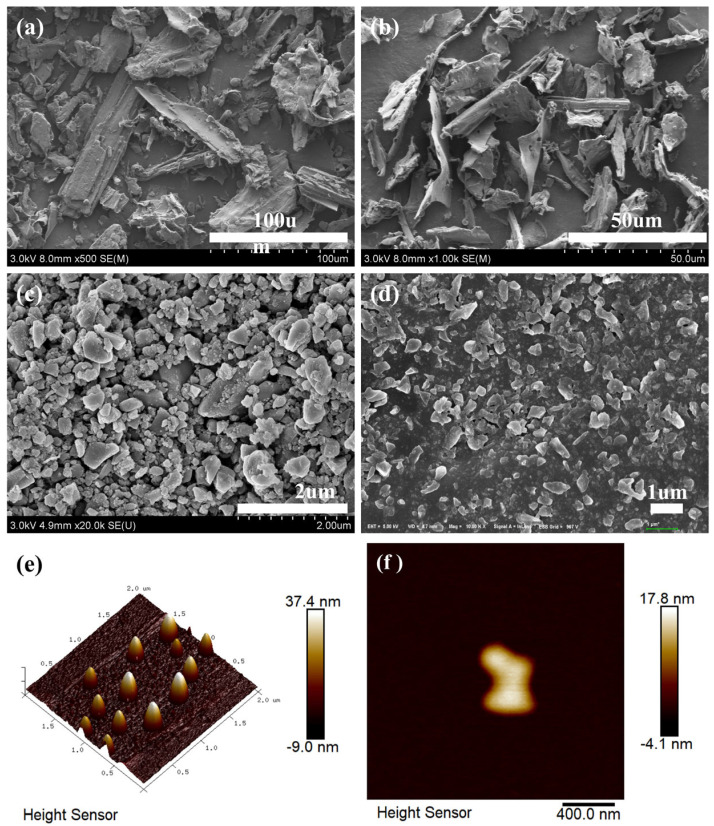
SEM of (**a**) biomass, (**b**) BC, (**c**) dried NBC, (**d**) NBC in ethanol; AFM of (**e**) NBCs, (**f**) NBC.

The specific surface area of the BC and NBC are 12.16 and 198.32 m^2^/g, respectively. The average pore size of the BC and NBC are 6.58 and 5.77 nm, respectively. This is mainly because the HPH process destroys the surface structure of biochar, thereby increasing the specific surface area of biochar. The sesame straw biomass powder is shown in Figure 2a, which exhibits a blocky or rod-like structure. The large particles of the biomass are sized at about 80 μm and are interspersed with small particles in the size of 10 μm. The BC is shown in Figure 2b, which has fewer three-dimensional structures and primarily exists in the form of flakes in the size of about 40 μm. The morphology of the NBC dissolved in ethanol is sized at 500 nm, exhibiting a relatively regular blocky structure with smoother edges. It is confirmed that the application of HPH technology can effectively reduce the particle size of biochar. During the HPH process, the biochar solution was strongly squeezed into a very narrow gap, and the biochar particles were subjected to extremely strong extrusion, high shear force, and impact force on the valve seat surface. In addition, the pressure dropped dramatically as the liquids flew through the narrow gap, creating numerous bubbles. Then, the pressure increased instantaneously as the liquids flew out of the narrow gap, leading to the rupture of bubbles. The cavity effect and high-frequency oscillation generated in this process also contributed to the fragmentation of the biochar particles. 

The dried NBC samples, as depicted in Figure 2c, predominantly possessed a flaky structure with a size of several hundred nanometers. However, serious agglomeration and stacking were observed in this NBC. In comparison to dried NBC, the NBC in ethanol is shown in Figure 2d, which achieved a more homogeneous dispersion state. The main reason for this result is the improved hydrophilicity of the biochar particles after alkaline modification bolstered the dispersion stability in ethanol. Consequently, it can be inferred that NBC should have good dispersion stability in EG with more hydroxyl groups. The AFM images of the biochar are presented in Figure 2e,f. The particle size of the NBC in Figure 2e is approximately 300 nm, with a maximum vertical height of about 37.4 nm. The particle size of the NBC depicted in Figure 2f is around 400 nm, and its thickness is approximately 17.8 nm. This confirms that the size of the NBC in one dimension is below 100 nm.

**Figure 3:**
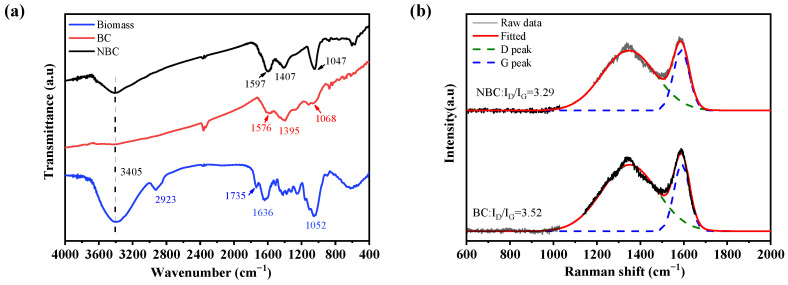
(**a**) FTIR spectra of biomass, BC, and NBC and (**b**) Raman spectra of pristine BC and NBC.

The FTIR spectra of the biomass powder, BC, and NBC are presented in Figure 3a. The peak around 3404 cm^−1^ corresponds to the stretching vibration of -OH [33]. In the spectra of the biomass powder, the peak of 2923 cm^−1^ corresponds to the stretching vibration of CHn [34], and the peak at 1735 cm^−1^ is assigned to the stretching vibration of carboxylic C=O [35], and the peak at 1636 cm^−1^ stands for the stretching vibration of C=O or C=C in the skeletal structure on the aromatic benzene ring [36]. In the fingerprint region, the peaks at 1376 cm^−1^ and 1052 cm^−1^ are ascribed to the bending vibration of CHn [37] and the stretching vibration of C-O [38], respectively. The peaks at 1576, 1395, and 1068 cm^−1^ in the FTIR spectra of the BC are attributed to the stretching vibrations of C=C on the aromatic ring, the bending vibration of -CHn, and the stretching vibration of C-O, respectively [39]. In the FTIR spectra of the NBC, the peak at 1597 cm^−1^ reflects the stretching vibration of C=O or C=C on the aromatic ring, while the peaks at 1395 and 1068 cm^−1^ refer to the bending vibration of -CHn and stretching vibration of C-O, respectively. 

The FTIR results indicate the contents of -OH- and other oxygen-containing functional groups significantly reduced during the pyrolysis of the sesame stalk biomass, while the aromatic content increased. A comparison of FTIR spectra between the BC and NBC reveals that the contents of -OH- and oxygen-containing functional groups significantly increased after the alkalization treatment, which is in line with the research by [40]. Hence, alkalinization treatment can increase the content of oxygen-containing functional groups in biochar. 

The Raman spectra of the pristine BC and NBC are shown in Figure 3b. The G peak at 1590 cm^−1^ corresponds to the in-plane vibration of the sp2 C atom, indicating the graphitization degree of the material, while the D peak at 1350 cm^−1^ corresponds to the defects in the structure of the carbon material [41]. The intensity ratio of the D peak to the G peak reflects the defect density in the carbon material. Compared to the BC, the NBC has a smaller particle size, which significantly increases the defects of C atoms at the boundaries. Additionally, the XPS and FTIR results indicate that the NBC introduced more oxygen-containing functional groups, which also caused more defects in the C atoms. However, according to the Raman results, the NBC exhibited less defect density than the BC, indicating the internal graphitization degree within the structure of the NBC is higher than that of the pristine BC. This is primarily due to the fact that the initially porous and collapsed structure of the BC transformed into a more compact block structure during the HPH process, enhancing the graphitization degree of the biochar.

**Figure 4:**
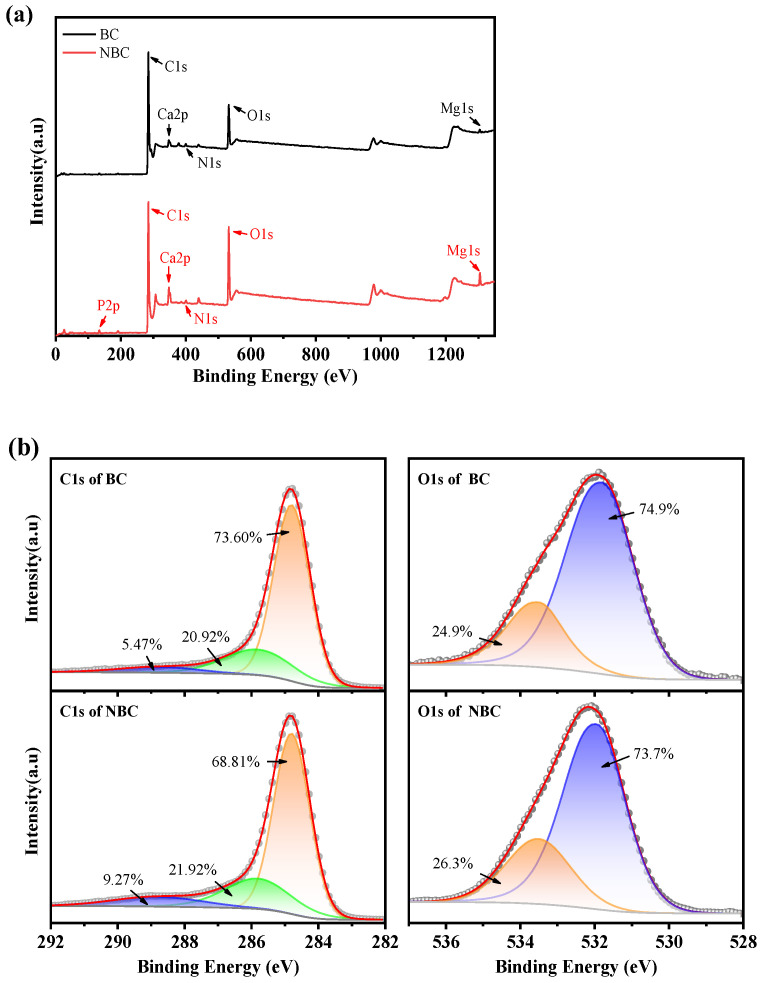
XPS of BC and NBC. (**a**) Total spectrum; (**b**) O1s and C1s spectrum.

The XPS results of the pristine BC and NBC are shown in Figure 4a. The surface elements of both the biochar samples primarily include C, O, N, P, Ca, and Mg, with the atomic fraction of each element listed in Table 1. The contents of Ca and Mg on the surface of the NBC sample are higher than in the BC, which is attributed to the fact that the Ca^2+^ and Mg^2+^ tend to precipitate in alkaline environments. In addition, the oxygen content on the surface of the NBC rose from 13.7% to 19.9% and carbon content decreased slightly compared to the BC.

The high-resolution O1s and C1s XPS spectra of the BC and the NBC are shown in Figure 4b. The C1s peaks of the BC are decomposed in C-C, C-O, and COO, with binding energy at 284.8, 285.81, and 288.72 eV, respectively [42]. Similarly, the deconvoluted C1s of the NBC peaks appear at 284.8, 285.81, and 288.80 eV, corresponding to C-C, C-O, and C=O, respectively [43,44]. Compared with the XPS result of the BC sample, the content of C-C decreases and the proportions of C-O and C=O increase in the NBC. 

In the O1s spectrum of the BC, the peaks at 531.75 and 533.5 eV are attributed to C-O and C=O, respectively, while the O1s spectrum of the NBC is decomposed into C-O (531.95 eV) and C=O (533.5 eV) [45]. The percentages of C-O and C=O are basically equal for both samples. The XPS results indicate the number of oxygen-containing functional groups in the modified NBC increased, with higher levels of C-O and C=O compared to the original BC, which is consistent with the FTIR results above.

### 3.2. Dispersion Stability of NBC Coolant

The equivalent diameters of the NBC particles under various homogenization conditions were measured using DLS. Owing to its excessively large particle size, the BC sample was unsuitable for the DLS measurement. To ensure that the test samples can exhibit the Tyndall effect and meet the requirements for the DLS test, the NBC powder was diluted with deionized water to a concentration of 0.001 wt.% and ultrasonically dispersed for 5 min. During the test, the temperature was maintained at 25 °C. The Baseline Index (BI) value of all the test results exceeded 6.5 (BI ≥ 5 is valid), indicating that the NBC sample met the testing requirements of DLS. 

**Figure 5:**
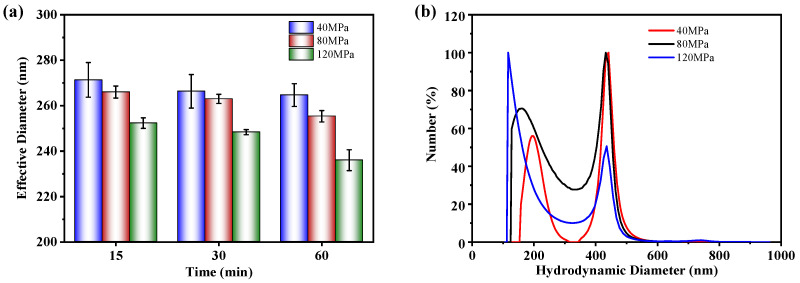
(**a**) Equivalent diameters of NBC at different HPH conditions; (**b**) particle size distribution of NBC at different homogenization pressures for 15 min.

The equivalent diameters of the NBC particles at different times and homogenizing pressures are depicted in Figure 5a. The influence of homogenization time on particle size was not obvious under the homogenization pressure at 40 MPa. Under the homogenizing pressure at 80 and 120 MPa, only homogenization time reaching 60 min led to a reduction in the particle size of the NBC. However, the particle size of the NBC reduced with the increasing homogenization pressure under the same homogenization time, indicating the homogenization pressure exerted a more pronounced effect on reducing the equivalent diameter of the NBC compared to the homogenization time. This could be explained by the fact that higher homogenization pressures imply stronger mechanical forces, leading to a reduction in the equivalent diameter of NBC particles [46].

Figure 5b demonstrates the particle size distribution of the NBC after 15 min of HPH under different pressures. Notably, the particle size distributions under different homogenization pressures all show bimodal characteristics, which is consistent with the SEM results above. For the smaller particles, the peaks are roughly located between 160 and 200 nm, and decrease with the increase in homogenization pressure while the peaks of larger particles are located around 440 nm under different homogenization pressures. 

**Figure 6:**
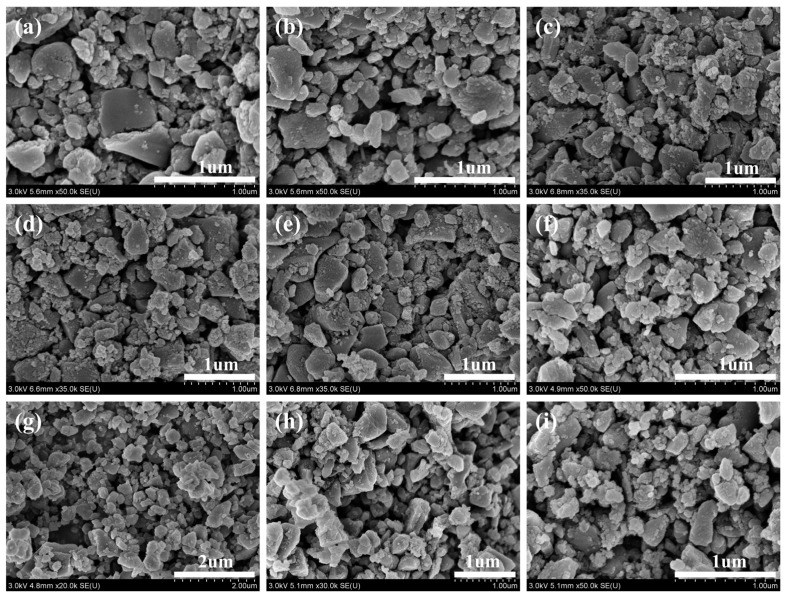
SEM of NBC at different HPH operation conditions: (**a**) 40 MPa, 15 min; (**b**) 40 MPa, 30 min; (**c**) 40 MPa, 60 min; (**d**) 80 MPa, 15 min; (**e**) 80 MPa, 30 min; (**f**) 80 MPa, 60 min; (**g**) 120 MPa, 15 min; (**h**) 120 MPa, 30 min; (**i**) 120 MPa, 60 min.

The SEM images under different HPH conditions are shown in Figure 6. Higher-resolution SEM images reveal that small particles consist of smaller primary particle clusters, and present a spherical and flower-like structure, while larger particles predominantly exhibit a blocky structure. The primary particles may be formed from the condensation of organic matter on the surface of biochar during the pyrolysis. Larger particles were mainly formed under the physical force during the HPH process, leading to the formation of nano-biochar. 

As shown in Figure 6a–c, under the homogenization pressure of 40 MPa, there were some larger biochar particles with diameters of about 600–700 nm, and the proportion of large particle biochar decreased as the homogenization time increased. As the homogenization pressure rose to 80 MPa, depicted in Figure 6d–f, the size of the larger particles slightly decreased, predominantly about 600 nm. As the homogenization pressure further rose to 120 MPa, depicted in Figure 6g–i, the particle size of the NBC samples became more uniform and smaller. Overall, with the increase in homogenization pressure and time, the size of the biochar particles became smaller and more uniform. Nevertheless, the impact of homogenization pressure is more pronounced than that of homogenization time. The size of biochar can be effectively reduced after HPH, which facilitates the stable dispersion of NBC in the EG.

**Figure 7:**
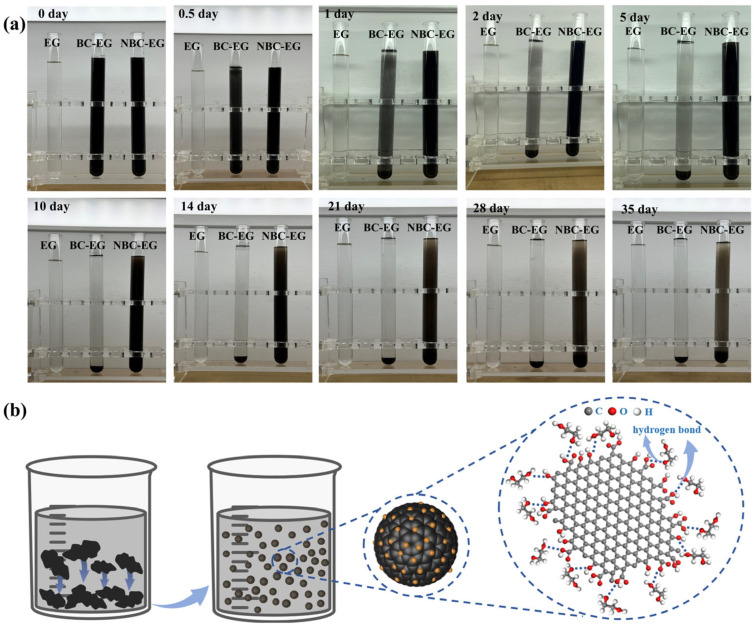
(**a**) Digital pictures of the samples; (**b**) schematic diagram of the dispersion stability of the NBC in EG.

The agglomeration and sedimentation of nanomaterials in nanofluids directly impact their thermal conductivity and application prospects, so it is essential to study the dispersion stability of nanofluids. The dispersion stability of 0.5 wt.% BC and 0.5 wt.% NBC in EG was assessed through the natural sedimentation method. The results were presented in Figure 7a, with the samples arranged from left to right as pure EG, BC-EG, and NBC-EG, successively. In the initial stage, both the BC and NBC were uniformly dispersed in EG. After 12 h, layering started at the supernatant of the BC sample, indicating that the BC particles began to agglomerate and precipitate in EG.

One day later, an obvious sedimentation phenomenon was observed in the BC sample, as most of the particles settled down to the tube bottom and only a few particles floated in the tube. After two days, the majority of the BC particles settled down, and a clear stratification was observed at the tube bottom. Five days later, the BC sample was completely sedimented, as the upper liquid layer was transparent and matched the color of pure EG. However, for the NBC sample, the particles still exhibited excellent dispersion stability in EG at the same time. By day 10, slight pre-stratification was observed in the uppermost layer of the NBC sample, indicating that the nanofluids were still stable. After 2 weeks, a more pronounced stratification occurred in the uppermost layer of the NBC sample, with the color of the uppermost liquid layer turning gray. Three weeks later, the grayish layer in the uppermost of the tube increased, indicating particles agglomerate or precipitate in the supernatant. The color of the NBC sample gradually turned gray and the particle settled down to the tube bottom after 4 weeks. After 5 weeks, the majority of the liquid in the upper part of the tube turned gray, and a significant amount of the NBC settled to the bottom.

The schematic diagram of the dispersion stability of the NBC in EG is shown in Figure 7b; NBC shows good dispersion stability in EG. This is mainly because NBC has a smaller size and larger surface area than BC, which can adsorb EG molecules in the surrounding environment to form a stable surface layer and reduce the tendency of particle aggregation and agglomeration. In addition, NBC possesses more oxygen-containing functional groups, such as carboxyl and hydroxyl groups, which can form hydrogen bonds with the hydroxyl groups in EG, thereby enhancing its binding strength in polar solvents such as EG.

### 3.3. Viscosity Analysis of NBC Coolant

**Figure 8:**
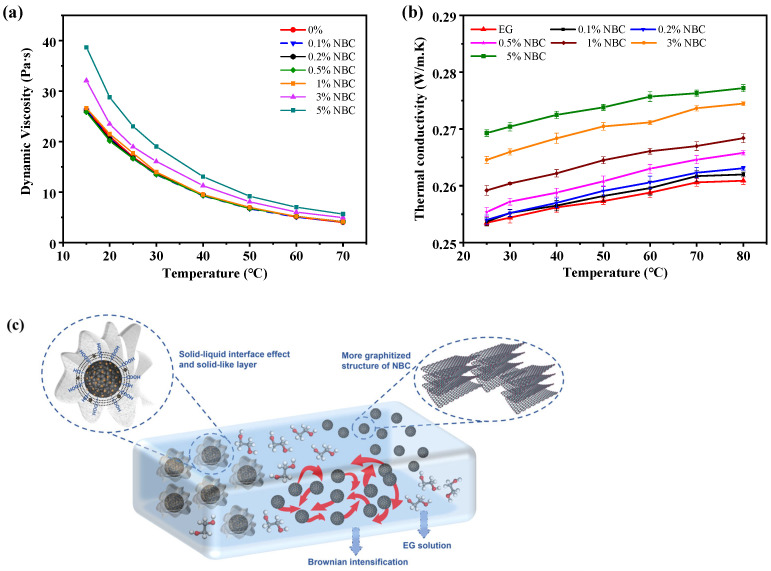
(**a**) Viscosity characteristic of NBC-EG at different temperatures, (**b**) the thermal conductivity/temperature characteristics of NBC-EG, and (**c**) the mechanisms of the NBC enhancing the thermal conductivity of EG.

As a critical parameter for coolants, dynamic viscosity directly influences the pumping efficiency of the cooling system. The viscosity/temperature characteristics of coolants were evaluated using a cone/plate viscometer. Figure 8a illustrates the viscosity/temperature characteristic curves of the NBC-EG solutions with different concentrations. The dynamic viscosity of all the samples decreases significantly as the temperature increases. When the NBC concentration is below 0.5 wt.%, the viscosity of the NBC-EG coolant nearly equals that of pure EG. At an NBC concentration of 1 wt.%, the viscosity of NBC-EG increased by 2.4% to 4.4% at lower temperatures compared with pure EG. As the temperature exceeds 40 °C, the viscosity is almost the same with pure EG. The main reason for this result is that the intermolecular forces between nanomaterials inside the nanofluids at higher temperatures are weakened, and the viscosity of the nanofluids decreases and approaches constant [47]. However, when the concentration of the NBC reaches 3 wt.%, the viscosity substantially increases with an average of 17% compared to pure EG, and 5 wt.% NBC concentration corresponded to an increase of an average of 38% in viscosity.

### 3.4. Thermal Conductivity of Coolants

The thermal conductivity of the NBC-EG solutions with different concentrations at varying temperatures is shown in Figure 8b. As the temperature rises, the thermal conductivity of all the samples is improved. This trend is primarily attributed to the accelerated molecular Brownian motion by the temperature rise, which enhances the frequency of energy exchange between atoms and thereby elevates the thermal conductivity of EG. 

The addition of 0.1 wt.% or 0.2 wt.% NBC does not significantly improve thermal conductivity. However, when the NBC concentrations are above 0.5 wt.%, the thermal conductivity is obviously enhanced. The addition of 0.5, 1, 3, and 5 wt.% NBC increases the thermal conductivity by 1.32%, 2.56%, 4.83%, and 6.23% compared to pure EG, respectively. The mechanisms of the NBC enhancing the thermal conductivity of EG are shown in Figure 8c. The thermal conductivity of pure biochar shall be close to that of graphite and carbon fiber (25–180 W/(m·K)) [48], which may be one reason for the improvement of the thermal conductivity. In addition, the Brownian motion of nanomaterials in nanofluids also contributes to the enhanced thermal conductivity of nanofluids. The irregular Brownian motion of nanomaterials improves the heat transfer rates and opportunity for collisions between particles, and between particles and liquids, further improving the heat transfer opportunity and the thermal convection within the heat transfer medium. Another possible mechanism is the liquid stratification at the liquid/particle interface. It is known that near the surface of particles, liquid molecules tend to form a layered structure similar to that of solids. The solid-like structure may enhance the thermal conductivity of nanofluids by providing thermal bridges between particles and liquids.

**Figure 9:**
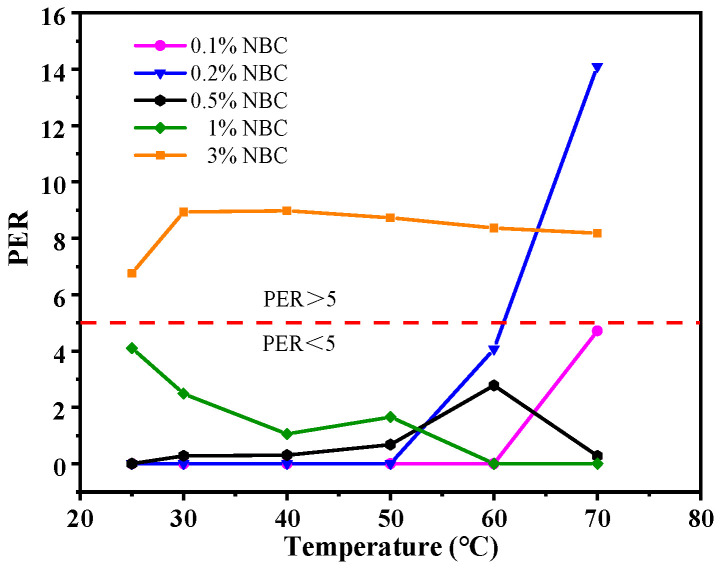
*PER* of NBC-EG at various temperatures.

Moreover, the enhancement of thermal conductivity is increased with increasing temperature. At 80 °C, the NBC nanofluids with 0.5, 1, 3, and 5 wt.% concentrations exhibited higher thermal conductivity compared to pure EG, with thermal conductivity enhancements of 1.88%, 2.87%, 5.20%, and 6.72%, respectively. This is mainly because the Brownian motion of the NBC is intensified with the rising temperature, enhancing the heat flux density and amplifying heat transfer of the nanofluids. However, when the NBC concentration is at 3 wt.% and 5 wt.%, the increase in the thermal conductivity of nanofluids is less than the three- or five-fold level of 1 wt.% NBC which is attributed to the agglomeration effect when the NBC concentration exceeds a certain threshold. Beyond that threshold, when the concentration exceeds a certain threshold, the increase in viscosity affects the pump efficiency and negatively impacts the energy consumption of the powertrain system. Therefore, the viscosity/temperature characteristics and thermal conductivity of the NBC-EG coolant shall be comprehensively considered. The performance enhancement ratio (*PER*), proposed by Prasher et al. [49], was used to evaluate the trade-off between the thermal conductivity and viscosity of nanofluid. the *PER* is calculated using Equation (1). According to the research by Garg, J [50], the nanofluid was suitable for heat transfer applications in a turbulent flow when the PER < 5; if the *PER* value is negative, it is considered to be zero.

(1) $$PER=\frac{\frac{\mu_{nf}}{\mu_{bf}}-1}{\frac{k_{nf}}{k_{bf}}-1}$$

where *µ_nf_* and *µ_bf_* are the viscosity of nanofluid and base fluid, respectively. *k_nf_* and *k_bf_* are the thermal conductivity of nanofluid, respectively. The *PER* values of the nano-biochar coolant at various temperatures are depicted in Figure 9. At a 0.1 wt.% of NBC concentration, the *PER* of the nanofluid meets the required standards at all temperatures. Upon increasing the concentration to 0.2%, there is a sharp increase in the *PER* value at 70 °C, primarily due to an increase in viscosity at high temperatures. As the concentration further increases to 0.5% and 1%, the nanofluid still meets the requirements at all temperatures. Yet, when the concentration reaches 3%, the sharp rise in viscosity, without a significant increase in thermal conductivity, results in an average *PER* value of 8.3 at all temperatures, which is no longer applicable as a coolant. For practical applications, the appropriate concentration of NBC shall be selected on the basis of working condition requirements.

Previous studies on the thermal conductivity of nanofluids and biochar-based compositions are listed in Table 2. Biochar is primarily utilized as an additive to enhance the thermal conductivity of phase change materials. In this application, the biochar content is high, with less regard for its dispersion stability and viscosity properties. In cooling media such as water or EG, the addition of nanomaterials enhances thermal conductivity, but also increases the viscosity. Considering its dispersibility stability, thermal conductivity, and viscosity properties, nano-biochar is a promising and environmentally friendly additive for enhancing the thermal conductivity of EG.

## 4. Conclusions 

In this study, the micro-sized sesame straw biochar was successfully converted into nano-biochar using the HPH method, the results show that the homogenization pressure more significantly impacts the size of biochar compared with homogenization time. The biochar samples were characterized using FTIR, Raman, and XPS. Compared to the BC, the NBC has more oxygen-containing functional groups, smaller size, and more graphitic structures, which contribute to the stable dispersion of the NBC in EG for 28 days without any additive. When 5 wt.% NBC is added to EG, the thermal conductivity of EG increases by 6.72%. This is mainly attributed to the graphitized structure and Brownian motion of the NBC. The application of green NBC materials in thermal management systems is significant for waste biomass recycling, carbon sequestration, and energy saving.

## Figures and Tables

**Table 1:** XPS elemental analysis of biochar surface.

Element/%	C	O	N	Mg	Ca	P
BC	81.0	13.7	2.1	0.6	1.7	0.9
NBC	73.4	19.9	1.4	1.5	2.6	1.2

**Table 2:** Summary of previous studies on the thermal conductivity of nanofluids and biochar-based composition.

Reference	Materials/Concentration	Base Fluid	Dispersity Stability	Viscosity	Enhancement of Thermal Conductivity
[11]	Carbon black (30 nm)/2 wt.%	EG			4.8%
	Carbon black (13 nm)/2 wt.%	EG			1.21%
[9]	Nano Al_2_O_3_/0.7 vol%	Water			4.2%
[1]	M-Ti_3_C_2_T_x_ MXene/5 vol%	EG		400%	53.1%
	S-Ti_3_C_2_T_x_ MXene/5 vol%	EG	30 days	76%	64.9%
[51]	Nano ZnO/3.5 wt.%	EG		15%	5.2%
	Nano ZnO/7 wt.%	EG		50%	9.13%
[23]	Bamboo biochar/(14.3 wt.%)	Stearic acid			5.2%
	Pine biochar/(14.3 wt.%)	Stearic acid			2.1%
	Walnut biochar/(14.3 wt.%)	Stearic acid			8.6%
	Corncob biochar/(14.3 wt.%)	Stearic acid			1.0%
[22]	Biochar (40 wt.%)	PCM (OM35)			22%
[24]	Garlic stem biochar/(5 wt.%)	Paraffin wax			7.2%
This study	Nano-biochar/(3 wt.%)	EG	28 days	17%	5.2%

## Data Availability

The data are available from the corresponding authors upon reasonable request.
